# Health literacy: a critical concept for public health

**DOI:** 10.1186/2049-3258-72-10

**Published:** 2014-04-01

**Authors:** Stephan Van den Broucke

**Affiliations:** 1 Psychological Sciences Research Institute, Université catholique de Louvain, Place Cardinal Mercier 10, 1348 Louvain-la-Neuve, Belgium

## 

Health literacy is gaining critical importance in public health. Since its introduction in the 1970s, the scientific literature on this subject has grown exponentially. To date, already more than 3000 publications on the topic are listed in Pubmed, 75% of which have been published in the last five years, and nearly 1000 have “health literacy” as a major MeSH term.

Originally, the interest in health literacy was mainly focused on health care services, and had a limited focus on the ability to handle words and numbers in a medical context. Yet over the years the concept gradually expanded in meaning to also account for more complex and interconnected abilities, such as reading and acting upon written health information, communicating needs to health professionals, and understanding health instructions [1,2]. More recently, the concept of health literacy has also found its way into public health. So, in addition to the already significant body of literature linking low health literacy to decreased medication adherence, poor knowledge of disease, poor adherence to self-care management, and poor treatment outcomes, there is now an increasing number of studies attesting to the fact that people with lower health literacy are also less likely to engage in health promoting behaviours [3], to participate in screening programs [4,5] or to use preventive services [6]. At the same time, the meaning of the concept itself continues to expand and now includes information seeking, decision making, problem solving, critical thinking, and communication, along with a multitude of social, personal, and cognitive skills that are imperative to function in the health system. This expansion, both in scope and in meaning, is well captured by the definition proposed by Sørensen et al. [1], which states that health literacy “*entails people’s knowledge, motivation and competences to access, understand, appraise, and apply health information in order to make judgments and take decisions in everyday life concerning healthcare, disease prevention and health promotion to maintain or improve quality of life during the life course”*. The definition incorporates both the medical and public health perspectives on health literacy, and accounts for the knowledge and competences that are required to meet the complex demands of modern society with regard to being ill, being at risk for illness, and staying healthy.

If health literacy can be considered as a critical determinant of public health, what is then its current status in the population? The available evidence suggests that more people have limited health literacy than is often assumed. According to population data from the US, nearly half of the American adult population may have difficulties in acting on health information [7]. In Europe, findings from the recent European Health Literacy Survey [8] indicate that 12% of the people surveyed have inadequate general health literacy, and 35% have problematic health literacy. Although the prevalence of problematic health literacy varies widely between countries (between 2% inadequate health literacy in the Netherlands versus 27% in Bulgaria) and between groups within populations, it is clear that health literacy is not just a problem of a small minority [9].

To address this “health literacy epidemic” [10], action is needed to improve health literacy. A recent policy document issued by the European Regional Office of WHO [9] calls for action at different levels: to ensure better health communication through establishing health literacy guidelines; to create and strengthen health literacy–friendly settings; and to develop policies for health literacy at the local, national and international level. These actions should be integrated to empower and enable people to make sound health decisions in the context of everyday life: at home, in the community, at the workplace, in the health care system, in the educational system, in the marketplace, and in the traditional and social media. While the health sector can lead by example through the creation of health care settings that empower patients and promote and support health literacy, politicians, professionals, civil society and the private sector should all contribute to addressing the health literacy challenges. International and regional agencies such as WHO, the EU, and the UN Economic and Social Council can provide moral and political platforms for actions.

Such actions must build on research. While the growing body of research on health literacy has expanded the scope and depth of the knowledge base about health literacy, produced a number of sophisticated and well-validated measures of health literacy [11-13], and demonstrated the links between poor health literacy and a variety of health outcomes, important knowledge gaps remain. For instance, the links between health literacy and documented health disparities among population groups is not yet well understood. More comprehensive research is also needed to understand the gap between individual capacities and demands posed by society and the health care system, and to evaluate the impact on health literacy of novel communication techniques and devices, including ICT driven communication tools. Finally, the potential of health literacy to serve as a proxy outcome measure of health education could be further explored [14].

In a strategic document published ten years ago, the American Medical Association [7] recommended four areas for health literacy research: health literacy screening, improving communication with low-literacy patients, costs and outcomes of poor health literacy, and causal pathways of how poor health literacy influences health. All these topics remain very relevant to date. The special series on health literacy of the *Archives of Public Health* aims to contribute to the important and timely task of providing research support to addressing low health literacy in the population.
